# Sex steroid hormones are associated with mortality in COVID-19 patients: Level of sex hormones in severe COVID-19

**DOI:** 10.1097/MD.0000000000027072

**Published:** 2021-08-27

**Authors:** Ingeborg E. van Zeggeren, Anita Boelen, Diederik van de Beek, Annemieke C. Heijboer, Alexander P.J. Vlaar, Matthijs C. Brouwer

**Affiliations:** a Amsterdam UMC, University of Amsterdam, Department of Neurology, Amsterdam Neuroscience, Meibergdreef 9, Amsterdam, the Netherlands; b Amsterdam UMC, University of Amsterdam, Department of Clinical Chemistry/Endocrine Laboratory, Amsterdam Gastroenterology & Metabolism, Meibergdreef 9, Amsterdam, the Netherlands; c Amsterdam UMC, Vrije Universiteit, Department of Clinical Chemistry/Endocrine Laboratory, Amsterdam Gastroenterology & Metabolism, de Boelelaan 1118, Amsterdam, the Netherlands; d Amsterdam UMC, University of Amsterdam, Department of Intensive Care, Meibergdreef 9, Amsterdam, the Netherlands; e Full list of contributors in Acknowledgments section.

**Keywords:** COVID-19, estradiol, outcome, SHBG, testosterone

## Abstract

In patients with coronavirus disease 2019 (COVID-19), men are more severely affected than women. Multiple studies suggest that androgens might play a role in this difference in disease severity. Our objective was to assess the association between sex hormone levels and mortality in patients with severe COVID-19.

We selected patients from the Amsterdam University Medical Centers COVID-19 Biobank, in which patients admitted to hospital in March and April 2020, with reverse transcription-polymerase chain reaction proven severe acute respiratory syndrome-coronavirus-2 infection, were prospectively included. Specifically, we included postmenopausal women (>55 years) and age-matched men, with a mortality of 50% in each group. Residual plasma samples were used to measure testosterone, estradiol, sex hormone binding globulin (SHBG), and albumin. We investigated the association of the levels of these hormones with mortality in men and women.

We included 16 women and 24 men in March and April 2020 of whom 7 (44%) and 13 (54%), respectively, died. Median age was 69 years (interquartile range [IQR] 64–75). In men, both total and free testosterone was significantly lower in deceased patients (median testosterone 0.8 nmol/L [IQR 0.4–1.9] in deceased patients vs 3.2 nmol/L [IQR 2.1–7.5] in survivors; *P* < .001, and median free testosterone 33.2 pmol/L [IQR 15.3–52.2] in deceased patients vs 90.3 pmol/L [IQR 49.1–209.7] in survivors; *P* = .002). SHBG levels were significantly lower in both men and women who died (18.5 nmol/L [IQR 11.3–24.3] in deceased patients vs 34.0 nmol/L [IQR 25.0–48.0] in survivors; *P* < .001). No difference in estradiol levels was found between deceased and surviving patients.

Low SHBG levels were associated with mortality rate in patients with COVID-19, and low total and free testosterone levels were associated with mortality in men. The role of testosterone and SHBG and potential of hormone replacement therapy needs further exploration in COVID-19.

## Abstract 2

Supplemental Digital Content is available in the text

## 1 Introduction

As of July 1, 2021, severe acute respiratory syndrome-coronavirus-2 has caused over 4 million registered deaths worldwide since the beginning of the pandemic in December 2019, according to the World Health Organisation.^[1]^ Coronavirus disease 2019 (COVID-19) can cause severe infection of the lungs, often leading to acute respiratory distress syndrome for which treatment strategies are needed.^[2,3]^ There has been a discrepancy in severity of the infection and mortality between men and women, in which males are more severely affected.^[4–7]^ Multiple studies suggest that androgens might play a role in this difference in disease severity, although associations with high and low levels differ.^[8–11]^ A substantially high percentage of men with COVID-19 were found to suffer from androgenetic alopecia, or ‘male pattern baldness’, which is associated with high levels of dihydrotestosterone.^[8,11]^ On the other hand, in a cohort of patients with prostate cancer, patients who received androgen-deprivation therapy were found to have lower risk of contracting severe acute respiratory syndrome-coronavirus-2.^[9]^ A study in 31 male COVID-19 patients showed that low total and free testosterone were associated with poor outcome.^[10]^

Sex differences in immune responses are a known phenomenon, not only in COVID-19.^[6,12,13]^ Sex hormones are thought to regulate the immune response, with estrogens providing a protective effect by enhancing cell-mediated and humoral immunity.^[12,13]^ Testosterone, on the other hand, is generally considered to have an immunosuppressive effect.^[12,13]^ In critically ill patients, however, low testosterone levels are frequently found but the etiology and clinical significance of this finding is still poorly understood.^[14,15]^

We hypothesized that levels of sex steroid hormones may differ between patients with COVID-19 infections with good and poor outcome. Therefore, we aimed to examine the levels of sex steroid hormones in male and female COVID-19 patients and their potential association to mortality.

## 2 Methods

### 2.1 Patients

For this study we included patients from the Amsterdam University Medical Centers (Amsterdam UMC) COVID-19 Biobank. In this biobank all consecutive patients, admitted to the Amsterdam UMC in Amsterdam, The Netherlands, from March 2020 onward, with confirmed COVID-19, were prospectively included in the biobank. Any residual diagnostic material of included patients was stored in the biobank. Comprehensive data on medical history, signs and symptoms, laboratory and radiological results, medication and course of the disease were collected in an online database using the World Health Organisation case record form. For the purpose of this study we retrospectively selected patients from this biobank from March and April 2020, consisting of postmenopausal women (>55 years) and age-matched men, with a mortality of 50% in each group. Patients could be either admitted in the intensive care unit (ICU) or the COVID wards. Stored residual material of these patients was used to measure plasma levels of estradiol, testosterone, and sex hormone binding globulin (SHBG). We investigated the association with outcome in this cohort of adult men and women with COVID-19, and correlated them with inflammation markers such as C-reactive protein and number of leukocytes in blood and clinical indicators of disease severity.

### 2.2 Hormone measurements

Residual diagnostic material of all patients was stored in –80°C until further analysis. We used 400 μL EDTA plasma from the first available blood sample after the day of admission, with a sufficient amount of residual material. Levels of estradiol and total testosterone were measured using liquid chromatography-tandem mass spectrometry as described previously,^[16,17]^ and SHBG using an enzyme-linked immunoassay (Architect, Abbott Diagnostics). Albumin was measured to calculate free and non-SHBG bound testosterone (free + albumin bound testosterone, according to Vermeulen).^[18,19]^ Reference values were established in house; estradiol: <26 pmol/L in postmenopausal women and 12 to 136 pmol/L in men^[17]^; total testosterone: 0.3 to 1.6 nmol/L in postmenopausal women, and 9 to 30 nmol/L in men^[19]^; free testosterone: 2.3 to 26 pmol/L in postmenopausal women, 165 to 655 pmol/L in men^[19]^; SHBG: 18 to 114 nmol/L in postmenopausal women and 13 to 71 nmol/L in men. Concentrations below or above the limits were defined as low and high, respectively.

### 2.3 Statistical analysis

Statistical analyses were performed using IBM SPSS Statistics for Windows, version 26 (IBM Corp., Armonk, NY). In this pilot study 20 patients per group (deceased vs survivors) provided sufficient power (alpha = 0.05, beta = 0.87) to detect a >2-fold difference between group means of hormone levels. For the comparison of categorical variables chi-square or Fisher exact test were used, whereas for continuous variables the Mann–Whitney *U* test was used. Spearman rank correlation was used for correlation analyses. All tests were 2-tailed with a *P* value <.05 considered statistically significant. In case of missing data, patients were left out of the analysis on that specific variable.

### 2.4 Ethics statement

This study was approved by the Biobank Ethical Review Committee of the Amsterdam UMC.

## 3 Results

We included 40 patients admitted to the Amsterdam UMC with confirmed COVID-19 in March and April 2020 consisting of 16 women and 24 men. The median age was 69 years (interquartile range [IQR] 64–75; Table 1). We found differences between men and women for ethnicity and rate of obesity (body mass index of ≥30) in which more men were white and women of African descent, while more women were obese (56% vs 21%, *P* = .02; Table 1). Laboratory chemistry parameters were comparable between sexes (Table 1). To our knowledge, no patients with known hypogonadism were included in the study. Out of the 40 patients, 15 (38%) were admitted to the ICU, of which 14 (34%) required mechanical ventilation. None of the patients received corticosteroids during admission. Out of all 40 patients, 20 patients died during admission (50%).

**Table 1:** Baseline characteristics included patients.

Characteristics	All patients (n = 40)	Women (n = 16)	Men (n = 24)	*P* value
Age (years)	69 (64–75)	68 (63–74)	71 (66–78)	.17
Ethnicity				
Arab	1/40 (3%)	1/16 (6%)	0/24 (0%)	.4
Black	5/40 (13%)	5/16 (31%)	0/24 (0%)	**.007**
South Asian	1/40 (3%)	1/16 (6%)	0/24 (0%)	.4
West Asian	1/40 (3%)	1/16 (6%)	0/24 (0%)	.4
Latin America	2/40 (5%)	2/16 (13%)	0/24 (0%)	.15
White	27/40 (68%)	3/16 (19%)	24/24 (100%)	**<.001**
Co-morbid conditions				
Chronic heart disease	12/40 (30%)	3/16 (19%)	9/24 (38%)	.18
Hypertension	22/40 (55%)	11/16 (69%)	11/24 (46%)	.13
Chronic neurological disorder	8/40 (20%)	1/16 (6%)	7/24 (29%)	.08
Obesity	14/40 (35%)	9/16 (56%)	5/24 (21%)	**.02**
Diabetes with complications	2/40 (5%)	0/16 (0%)	2/24 (8%)	.51
Diabetes w/o complications	10/40 (25%)	5/16 (31%)	5/24 (21%)	.48
Laboratory results				
White blood cell count (10^9^/L)	7.2 (6.2–11.0)	7.2 (6.1–10.5)	7.1 (6.0–12.9)	.94
Lymphocyte count (10^9^/L)	0.9 (0.72–1.2)	0.89 (0.74–1.4)	0.92 (0.68–1.2)	.90
Neutrophil count (10^9^/L)	5.7 (4.6–9.2)	5.85 (4.8–9.0)	5.4 (4.5–11.4)	.84
Platelets (10^9^/L)	231 (191–279)	236 (206–288)	223 (169–261)	.24
PT (seconds; n = 11)	11.5 (10.7–12.5)	11.5 (10.8–28.8)	12.0 (10.6–12.6)	.93
aPTT (seconds; n = 14)	29.0 (27.8–33.8)	26.0 (24.0–33.0)	30.0 (26.5–34.5)	.71
ALAT (units/L)	23.5 (16.3–38.0)	21.5 (15.5–36.8)	26.0 (17.3–47.0)	.60
ASAT (units/L)	38.0 (30.0–55.5)	42.0 (32.5–55.5)	36.5 (26.3–58.0)	.46
Total bilirubin (μmol/L)	9.0 (7.0–10.0)	8.0 (5.3–10.0)	9.0 (7.5–11.0)	.20
Glucose (mmol/L)	7.5 (6.5–9.3)	7.3 (6.7–9.1)	8.0 (6.3–9.7)	.75
Creatinine (μmol/L)	90.0 (70.5–125.0)	76.0 (63.5–120.5)	92.0 (79.8–128.5)	.16
CRP (mg/L)	110.6 (71.1–189.7)	91.9 (65.8–117–5)	139.2 (82.1–198.1)	.085
CK (units/L)	123.5 (70.5–228.8)	206.0 (76.5–314.8)	103.0 (55.5–180.3)	.25
LDH (units/L)	428.5 (322.3–511.0)	469.5 (387.3–608.8)	371.5 (256.0–470.5)	.56
D-dimer (mg/L)	1.01 (0.51–1.87)	1.01 (0.61–2.09)	0.86 (0.30–1.87)	1.00
Admission details				
ICU admission	15/40 (38%)	4/16 (25%)	11/24 (46%)	.32
Invasive ventilation	14/40 (35%)	4/16 (25%)	10/24 (42%)	.33
Deceased	20/40 (50%)	7/16 (44%)	13/24 (54%)	.75

Values are n/N(%) or median (IQR). ALAT = alanine aminotransferase, aPTT = activated partial-thrombine time, ASAT = aspartate aminotransferase, CK = creatine kinase, CRP = C-reactive protein, ICU = intensive care unit, IQR = interquartile range, LDH = lactate dehydrogenase, PT = prothrombine time.

Median number of days from first disease symptom onset to the day of blood withdrawal was 11 (IQR 8.5–14), and the median number of days between admission and day of blood withdrawal was 2 (IQR 1–3). Median number of days from date of sample to date of death was 5 (IQR 2–9). No association was found between the hormone levels and day of acquisition of the sample (Spearman rank).

**Figure 1:**
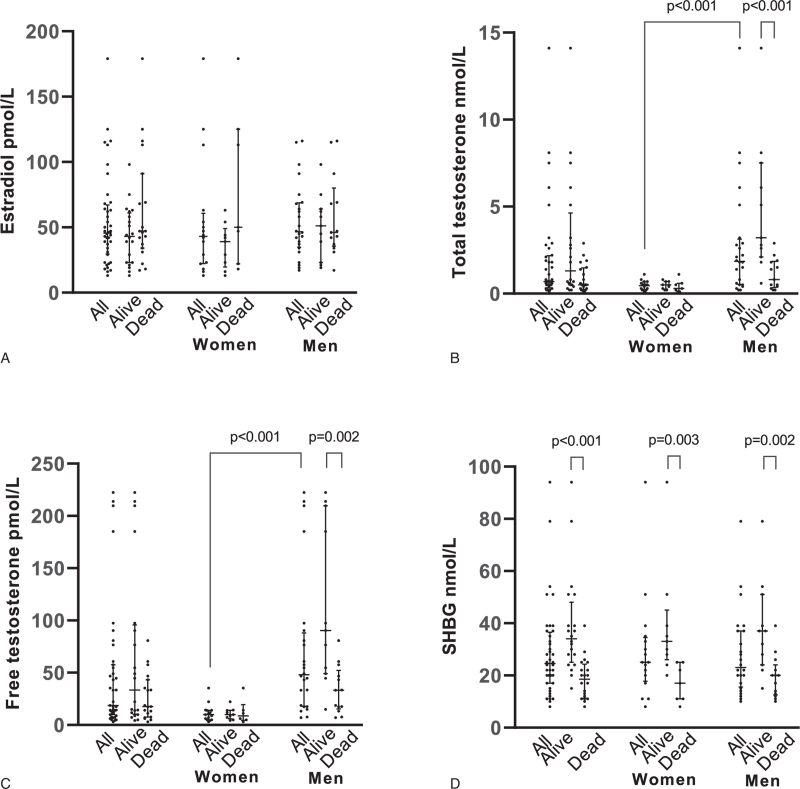
Sex hormone levels in all, surviving and deceased patients. A: estradiol levels in pmol/L, B: total testosterone levels in nmol/L, C: free testosterone levels in pmol/L, D: sex hormone binding globulin (SHBG) levels in nmol/L. For A to D: black dots are separate patients, black lines are medians with interquartile range.

The median level of estradiol was 43.0 pmol/L (IQR 22.3–60.8) in women and 46.5 pmol/L (IQR 34.8–106.5) in men (Fig. 1). All men had an estradiol level within normal range, whereas 69% (n = 11) of the women had estradiol levels above the reference range (Figure S1, Supplemental Digital Content, http://links.lww.com/MD2/A354). Estradiol levels were similar between surviving and deceased patients, with levels of 43.0 (IQR 23.0–61.0) and 47.0 (IQR 34.8–107.5), respectively (*P* = .165). This did not differ within the groups of just men and just women (Fig. 1).

Total testosterone levels were 0.45 nmol/L (IQR 0.2–0.68) in women and 1.85 nmol/L (IQR 0.53–3.1) in men. The median level of free testosterone in women was 9.9 pmol/L (IQR 4.9–14.1) and 48.0 pmol/L (IQR 17.8–88.0) in men. All but 1 male patient (96%) had a total testosterone below the lower limit of the reference interval, of whom 20 (83%) also had a free testosterone below the lower limit of the reference interval. In men, total testosterone was lower in deceased patients, with a median level of 0.8 nmol/L (IQR 0.4–1.9) in deceased patients vs 3.2 nmol/L (IQR 2.1–7.5) in surviving patients (*P* < .001; Fig. 1). A similar association was found for free testosterone (*P* < .001).

Median level of SHBG in women was 25.0 nmol/L (IQR 17.8–34.5) and 23.0 nmol/L in men (IQR 15.5–37.0; *P* = .33). SHBG levels were lower in both male and female deceased patients compared to survivors, with a median SHBG level in women of 17.0 nmol/L (IQR 11.0–25.0) in deceased patients and 33.0 nmol/L (IQR 26.0–45.0) in survivors (*P* = .003); in men, this was 20.0 nmol/L (IQR 12.5–24.0) in deceased patients vs 37.0 nmol/L (IQR 24.0–51.0) in survivors (*P* = .002).

## 4 Discussion

Our study shows that lower levels of total and free testosterone were associated with fatal outcome in men who died of COVID-19, as were lower levels of SHBG in both men and women. Despite low and normal levels of SHBG, both total and free testosterone levels were low in almost all men, meaning that these men suffered from testosterone deficiency. A substantial proportion of women had a level of estradiol above the upper normal limit (69%), but this was not associated with disease outcome. These elevated estradiol levels might be attributable to the higher number of women with obesity in this group, since adipose tissue is a major source of estrogen production due to aromatization of both testosterone and androstenedione in these cells.^[20,21]^

Previously, in an Italian study with 31 hospitalized men with COVID-19, no significant differences between SHBG levels were identified in patients who were admitted to a regular ward compared to ICU or deceased patients.^[10]^ SHBG levels in these groups did, however, seem comparable to the levels we found.^[10]^ The mortality rate in the Italian study was substantially lower (13%) compared to our study (44% in men) which gave our study more power to detect significant differences. Several mechanisms may explain the association between SHBG and mortality. First, SHBG can be influenced by diet, body mass index, insulin levels, and age. Since patients with COVID-19 frequently have obesity and presumably higher levels of insulin due to insulin resistance, this might be a partial explanation for lower levels of SHBG.^[22]^ Additionally, hypoalbuminaemia due to increased vascular permeability and capillary leakage is common in critically ill patients,^[23]^ and a similar mechanism could lead to low SHBG concentrations. Finally, SHBG levels have also been associated with thyroid hormone levels.^[24]^ Patients in the ICU often have decreased levels of thyroid hormones, such as tri-iodothyronine and thyroxine, which is known as non-thyroidal illness syndrome and associated with a poor prognosis.^[25]^ This might also explain the lower SHBG levels we found, since low tri-iodothyronine levels have also been described in COVID-19 patients and were associated with severity of the disease.^[26,27]^

Low levels of plasma total and free testosterone have been described in critically ill men with severe infections, both severe acute respiratory syndrome-coronavirus-2 and other types of infections, with testosterone levels corresponding to our results.^[10,14,15]^ In women with COVID-19, differences in total testosterone between non-severe and severe COVID-19 patients were described as well, although most measurements were within the reference range.^[28]^ Low testosterone levels in acute critically ill patients are probably caused by down-regulation of the gonadal axis by cytokines or corticotropin releasing hormone and corticosteroids.^[29–31]^ In patients with chronic obstructive pulmonary disease, who often have testosterone deficiencies, hormone replacement therapy resulted in reduction of respiratory hospitalizations in certain groups.^[32]^ Whether hormone replacement therapies might have a role in COVID-19, either through a protective, anti-inflammatory effect or through its anti-catabolic effect on respiratory muscles, should be evaluated in future research.^[33]^ Furthermore, it is unclear whether testosterone levels were also low before initial infection or admission, and in order to proof causality pre-COVID-19 levels in prospectively collected samples would be needed.

Our study has several strengths and limitations. A strength of this study are the methods that were used for hormone measurements, which are very specific and sensitive in measuring low hormone concentrations like we found in our patients. A limitation is the retrospective design. Although clinical data were collected prospectively, both patients and samples that were used for this analysis were selected retrospectively. For the blood samples this was based on availability of sufficient amount of residual material. Because of this, used samples were withdrawn on different moments during admission, which makes it difficult to say anything about the course of the hormone levels. Also, we were unable to measure tri-iodothyronine due to insufficient amounts of blood left after hormone measurements. Furthermore, the small sample size makes it difficult to do multivariate logistic regression. However, this study was set up as a pilot and we were able to demonstrate significant differences in hormone levels between groups.

In conclusion, total and free testosterone levels in men, and SHBG levels in all patients, are associated with outcome of admitted patients with COVID-19. Whether these sex hormones influence the disease course or are mere markers of disease severity remains to be elucidated. Future research should focus on prospective measurements of sex hormone levels and the possible role of hormone replacement therapies in COVID-19.

## Acknowledgments

Amsterdam UMC COVID-19 Biobank authors: Michiel van Agtmael^2^, Anne Geke Algera^1^, Frank van Baarle^1^, Diane Bax^3^, Martijn Beudel^4^, Harm Jan Bogaard^5^, Marije Bomers^2^, Peter Bonta^5^, Lieuwe Bos^1^, Michela Botta^1^, Justin de Brabander^2^, Godelieve Bree^2^, Sanne de Bruin^1^, Marianna Bugiani^5^, Esther Bulle^1^, Osoul Chouchan^2^, Alex Cloherty^3^, Dave A. Dongelmans^1^, Paul Elbers^1^, Lucas Fleuren^1^, Suzanne Geerlings^2^, Bart Geerts^6^, Theo Geijtenbeek^3^, Armand Girbes^1^, Bram Goorhuis^2^, Martin P. Grobusch^2^, Florianne Hafkamp^3^, Laura Hagens^1^, Jorg Hamann^7^, Vanessa Harris^2^, Robert Hemke^8^, Sabine M. Hermans^2^, Leo Heunks^1^, Markus Hollmann^6^, Janneke Horn^1^, Joppe W. Hovius^2^, Menno D. de Jong^9^, Rutger Koning^4^, Niels van Mourik^1^, Esther J. Nossent^5^, Jeaninne Nellen^2^, Frederique Paulus^1^, Tom Reijnders^2^, Edgar Peters^2^, Tom van der Poll^2^, Bennedikt Preckel^6^, Jan M. Prins^2^, Jorinde Raasveld^1^, Michiel Schinkel^2^, Marcus J. Schultz^1^, Alex Schuurmans^10^, Kim Sigaloff^1^, Marry Smit^1^, Cornelis S. Stijnis^2^, Willemke Stilma^1^, Charlotte Teunissen^11^, Patrick Thoral^1^, Anissa Tsonas^1^, Marc van der Valk^2^, Denise Veelo^6^, Heder de Vries^1^, Michèle van Vugt^2^, Dorien Wouters^12^, A. H (Koos) Zwinderman^13^, Matthijs C. Brouwer^4^, W. Joost Wiersinga^2^, Alexander P.J. Vlaar^1^, Diederik van de Beek^4^

^1^Department of Intensive Care, Amsterdam UMC, Amsterdam, The Netherlands; ^2^Department of Infectious Diseases, Amsterdam UMC, Amsterdam, The Netherlands; ^3^Experimental Immunology, Amsterdam UMC, Amsterdam, The Netherlands; ^4^Department of Neurology, Amsterdam UMC, Amsterdam, The Netherlands; ^5^Department of Pulmonology, Amsterdam UMC, Amsterdam, The Netherlands; ^6^Department of Anesthesiology, Amsterdam UMC, Amsterdam, The Netherlands; ^7^Amsterdam UMC Biobank Core Facility, Amsterdam UMC, Amsterdam, The Netherlands; ^8^Department of Radiology, Amsterdam UMC, Amsterdam, The Netherlands; ^9^Department of Medical Microbiology, Amsterdam UMC, Amsterdam, The Netherlands; ^10^Department of Internal Medicine, Amsterdam UMC, Amsterdam, The Netherlands; ^11^Neurochemical Laboratory, Amsterdam UMC, Amsterdam, The Netherlands; ^12^Department of Clinical Chemistry, Amsterdam UMC, Amsterdam, The Netherlands; ^13^Department of Clinical Epidemiology, Biostatistics and Bioinformatics, Amsterdam UMC, Amsterdam, The Netherlands

## Author contributions

IvZ contributed to data gathering, data analyses, data interpretation, and writing of the first draft of the manuscript. AB, DvdB, AH and AV contributed to study design, data interpretation, review, and critique of the report. Authors of the AUMC COVID19-Biobank contributed to gathering of data. MB contributed to study design, data analysis, data interpretation, review, and critique of the report.

**Formal analysis:** Ingeborg van Zeggeren, Anita Boelen.

**Funding acquisition:** Matthijs C. Brouwer.

**Investigation:** Ingeborg van Zeggeren, Anita Boelen, Annemieke C. Heijboer.

**Methodology:** Anita Boelen, Diederik van de Beek, Annemieke C. Heijboer, Matthijs C. Brouwer.

**Supervision:** Anita Boelen, Diederik van de Beek, Annemieke C. Heijboer, Alexander P.J. Vlaar, Matthijs C. Brouwer.

**Writing – original draft:** Ingeborg van Zeggeren.

**Writing – review & editing:** Anita Boelen, Diederik van de Beek, Annemieke C. Heijboer, Alexander P.J. Vlaar, Matthijs C. Brouwer.
